# Toward standardized guidelines for distal perfusion cannulae in V-A ECMO and ECPELLA: timing, sizing, monitoring, and management

**DOI:** 10.1051/ject/2025065

**Published:** 2026-06-19

**Authors:** Salman Pervaiz Butt, Salman Abdulaziz, Nabeel Razzaq, Arun Kumar, Mehnaz Farheen, Rania Khalil, Vivek Kakar, Umer Darr, Gopal Bhatnagar

**Affiliations:** 1 Perfusion Services, HVTI, Cleveland Clinic PO BOX 112412 Abu Dhabi UAE; 2 Critical Care Services Administration, King Fahad Medical City Riyadh KSA; 3 Anesthesiology Institute, Cleveland Clinic PO BOX 112412 Abu Dhabi UAE; 4 Critical Care, Cleveland Clinic PO BOX 112412 Abu Dhabi UAE; 5 Cardiac Surgery, HVTI, Cleveland Clinic PO BOX 112412 Abu Dhabi UAE

Dear Editor,

Acute limb ischemia (ALI) remains one of the most common complications of peripheral veno-arterial extracorporeal membrane oxygenation (V-A ECMO). Across heterogeneous cohorts, reported ALI rates vary widely (≈8–30% and higher in selected series), reflecting differences in patient selection, cannulation technique, and monitoring practices. Despite mounting observational and meta-analytic evidence, there is still no unified, evidence-graded guidance on when and how to use a distal perfusion cannula (DPC). How to size, connect, monitor adequacy, and how to adapt protocols when V-A ECMO is combined with femoral Impella (ECPELLA) [1, 2]. Figure 1 shows the common DPC orientation.

**Figure 1:**
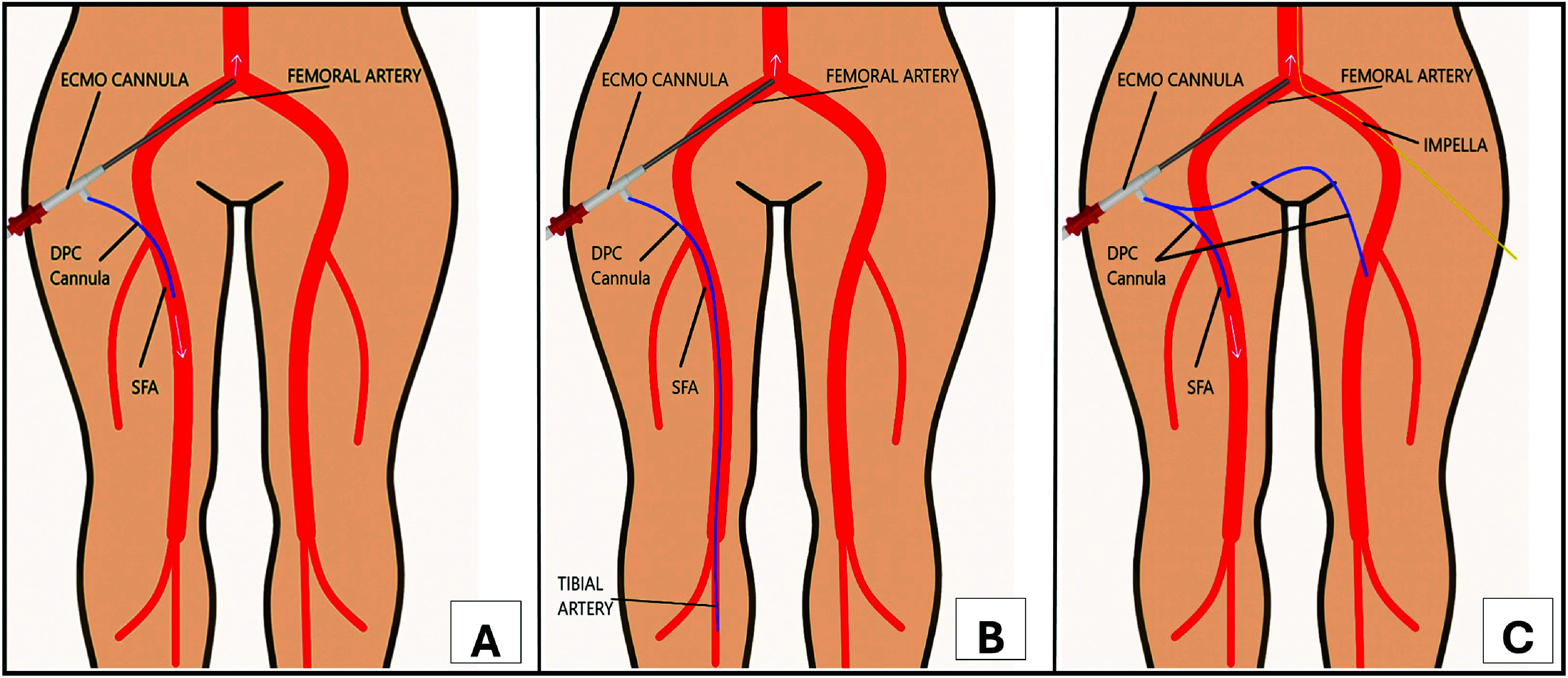
DPC cannula orientation towards; A: Superficial femoral artery, B: Tibial artery, C: In ECPELLA.

Evidence currently associates prophylactic DPC or the use of smaller-bore arterial return cannulae with lower odds of limb ischemia [3, 4]. Random-effects meta-analysis studies report risk reductions of ~60% with prophylactic DPC and ~60% with smaller return cannulae [2, 5]. Single-center and registry studies reinforce these findings and highlight the multifactorial nature of ALI (artery size relative to cannula, sex, age, atherosclerotic burden, shock severity, vasopressor dose, and decannulation technique) [1, 6, 7].

We propose that professional societies consider a consensus pathway with the following pragmatic pillars:

*Timing*: Early DPC placement- preferably at cannulation. Prophylactic placement protocols that embed prophylactic have reported marked reductions in ALI compared with reactive strategies [2].

*Size & circuit*: Use a reinforced 6–8 Fr antegrade sheath in the superficial femoral artery (SFA). Connected via short, small-diameter tubing to maintain adequate blood flow, reduce stasis, and minimize thrombosis risk within the DPC loop. Avoid unnecessarily large arterial cannula; target the lowest Fr size that achieves hemodynamic goals. Smaller cannula are associated with lower ALI risk [2, 5, 8].

*Placement technique*: Favor ultrasound-guided antegrade SFA cannulation; confirm position and runoff with Doppler or vascular ultrasound. Fluoroscopy-guided or hybrid approaches are reasonable when anatomy is uncertain or when reactive placement is required after angiography [9].

*Monitoring*: Implement tiered monitoring: Hourly bedside vascular checks for flow confirmation. Near-infrared spectroscopy (NIRS) with bilateral calf probes. Evidence suggests NIRS thresholds (e.g., absolute rSO_2_ <40% or ≥25% drop from baseline) can cue timely intervention and reduce the need for surgical rescue [1, 10–12].

*Escalation & rescue*: If hypoperfusion persists despite an optimally placed DPC, consider upsizing the DPC. Optimize ECMO flow and mean arterial pressure, relieving venous congestion, and consider early vascular surgery consultation. Percutaneous strategies, including temporary extracorporeal femoro-femoral crossover perfusion or radial-to-femoral external bypass, can rapidly restore antegrade flow when anatomy or devices preclude simple solutions [13–15].

*Decannulation & repair*: During decannulation, where percutaneous closure is used, assess for late stenosis/occlusion. Have a low threshold for duplex imaging [1, 6].

*ECPELLA*: ECPELLA use increases ischemia risk. Observational cohorts describe a simple, reproducible mitigation strategy: bilateral antegrade DPC use where one DPC is inserted distal to the ECMO cannula, and one distal to the Impella. Use of appropriate blood flow sources is needed, which is scarce during Impella use solely, highlighting a potential gap in care. Evidence has shown zero ischemic events in the center series when used prophylactically [12, 16]. If limb perfusion remains marginal, percutaneous contralateral crossover or external bypass can be lifesaving bridges until definitive reconfiguration (e.g., axillary Impella or upper-body cannulation) is achieved [13–15].

*Future considerations*: New bidirectional cannulae are emerging on the market to address ECMO-related limb ischemia, where dedicated DPC channels are integrated into the arterial cannula. Although not currently widely used, this is an area where innovation may occur.

In summary, a consensus, checklist-driven framework is urgently needed to standardize timing, size, connection, and monitoring of DPCs in femoral VA-ECMO with explicit algorithms for ECPELLA. Such guidance should be evidence-graded, adaptable to resource settings, and include audit metrics (e.g., DPC placement rate in eligible femoral V-A ECMO, NIRS adoption, ALI/fasciotomy/amputation rates, time-to-DPC). We invite a society-led, multi-disciplinary consensus and pragmatic trials to close remaining evidence gaps.

## Data Availability

No new data were generated or analyzed in this study. All data supporting the statements and recommendations in this article are derived from previously published literature, which is appropriately cited.

## References

[1] Bonicolini E, Martucci G, Simons J, et al. Limb ischemia in peripheral venoarterial extracorporeal membrane oxygenation: a narrative review of incidence, prevention, monitoring, and treatment. Crit Care. 2019;23(1):266. 31362770 10.1186/s13054-019-2541-3 PMC6668078

[2] Marbach JA, Faugno AJ, Pacifici S, et al. Strategies to reduce limb ischemia in peripheral venoarterial extracorporeal membrane oxygenation: a systematic review and meta-analysis. Int J Cardiol. 2022;361:77–84. 35523371 10.1016/j.ijcard.2022.04.084

[3] Lorusso R, Whitman G, Milojevic M, Raffa G, et al. 2020 EACTS/ELSO/STS/AATS expert consensus on post-cardiotomy extracorporeal life support in adult patients. Eur J Cardiothorac Surg. 2020;59(1):12–53. 10.1093/ejcts/ezaa283 33026084

[4] Geller BJ, Sinha SS, Kapur NK, Bakitas M, et al. Escalating and de-escalating temporary mechanical circulatory support in cardiogenic shock: a scientific statement from the American Heart Association. Circulation. 2022;146(6):e50–e68. 35862152 10.1161/CIR.0000000000001076

[5] Juo YY, Skancke M, Sanaiha Y, et al. Efficacy of distal perfusion cannulae in preventing limb ischemia during extracorporeal membrane oxygenation: a systematic review and meta-analysis. Artif Organs. 2017;41(11):E263–E273. 28762511 10.1111/aor.12942

[6] Hart JP, Davies MG. Vascular complications in extracorporeal membrane oxygenation: a narrative review. J Clin Med. 2024;13(17):5170. 39274383 10.3390/jcm13175170 PMC11396245

[7] Dragulescu R, Acalovschi I, Santoro A, et al. Lower limb ischemia in surgical femoral veno-arterial ECMO is uncommon and not associated with in-hospital mortality; female sex is a risk factor. J Vasc Surg. 2023;78(3):e258–e268.

[8] Anderson D, Burrell A, Collins S, Diehl A. Alfred ECMO Guideline: Distal perfusion cannula – choice, circuit, and monitoring. ecmo.icu. Published 2024. Available at: https://ecmo.icu.

[9] Jang WJ, Chun WJ, Choi TK, et al. Fluoroscopy-guided simultaneous distal perfusion as a rescue strategy for lower limb ischemia during femoral veno-arterial ECMO. Ann Intensive Care. 2018;8:38. 30374593 10.1186/s13613-018-0445-z PMC6206319

[10] Vinogradsky A, Kurlansky P, Ning Y, Kirschner M, et al. Continuous near-infrared reflectance spectroscopy monitoring to guide distal perfusion can minimize limb ischemia surgery for patients requiring femoral ECMO. J Vasc Surg. 2023;S0741-5214:20227367. 10.1016/j.jvs.2022.12.057 36603665

[11] Coelho R, Tavares J, Marinheiro C, et al. The effectiveness of NIRS technology for early diagnosis of lower limb ischemia in peripheral VA-ECMO: a systematic review and meta-analysis. Intensive Crit Care Nurs. 2025;89:104039. 40233544 10.1016/j.iccn.2025.104039

[12] Ortoleva J. Limb ischemia in femoral venoarterial ECMO patients: cutting to the chase? J Cardiothorac Vasc Anesth. 2023;37(11):2280–2281. 37635041 10.1053/j.jvca.2023.07.045

[13] Richarz S, Selch D, Neumann T, et al. Temporary extracorporeal femoro-femoral crossover bypass to prevent severe limb ischemia caused by Impella CP. J Vasc Surg Cases Innov Tech. 2022;8(1):63–67.

[14] Lichaa H, Khalife WI, Jordan M, et al. External radial-to-femoral bypass for antegrade perfusion to treat limb ischemia related to large-bore access. US Cardiol Rev. 2020;14:e01.

[15] Saito Y, Kobayashi Y, Takahashi K, et al. Complications and outcomes of Impella treatment in cardiogenic shock patients with and without acute myocardial infarction: a nationwide registry analysis. J Am Heart Assoc. 2023;12:e030819. 37646217 10.1161/JAHA.123.030819 PMC10547360

[16] Whitmore S, Castiglia R, Jarzembowski J, et al. Standard placement of bilateral distal perfusion catheters to prevent limb ischemia during femoral VA-ECMO plus Impella CP (ECPELLA). ASAIO J. 2024;70:23.

